# COVID-19: first and second wave impact on outpatient service users of FACT or autism teams in the Netherlands

**DOI:** 10.1192/j.eurpsy.2022.789

**Published:** 2022-09-01

**Authors:** S. Castelein, I.D.C. Van Balkom, J. Bruins

**Affiliations:** 1 Lentis Psychiatric Institute, Lentis Research, Groningen, Netherlands; 2 University of Groningen, Clinical Psychology, Groningen, Netherlands; 3 Lentis Psychiatric Institute, Autism Team Northern-netherlands, Jonx Department Of (youth) Mental Health And Autism, Zuidlaren, Netherlands

**Keywords:** Covid-19, TeleHealth, Psychosis, government measures

## Abstract

**Introduction:**

Most research on COVID-19 effects has focused on the general population. Here we measure its impact on Dutch FACT and autism outpatient service users during both waves.

**Objectives:**

This study aimed to: 1) investigate participants’ mental health, 2) assess experiences with outpatient services, and 3) assess respondents’ experiences with governmental measures in the Netherlands during the first and second wave of COVID-19.

**Methods:**

Respondents (wave 1: n=100; wave 2: n=150) reported on mental health, experiences with outpatient care, government measures and information services in an online survey.

**Results:**

Findings demonstrate happiness was rated an average of 6 out of 10, 70% of respondents scored below average on resilience, positive consequences for mental health (ordered world, reflection time) during both waves were similar, and prominent negative consequences included decreased social interactions and increased or new problems regarding mental health and daily functioning from wave 1-2. Lifestyle changed in 50% in both waves, although only slightly attributed to the pandemic. Substance use during both waves hardly changed. Mental healthcare continuation was highly appreciated in both waves (75-80% scored ≥7 on 10-point scale). (Video)calling was the most frequently mentioned positive care experience; missing face-to-face contact with care providers considered most negative. COVID-19 measures were less doable in the second wave. Vaccination willingness approximated 70%.

**Conclusions:**

Results show a nuanced, but clear picture of experiences during both waves. Continuation of services through tele-health was well-received. Monitoring of long-term impact is needed.

**Disclosure:**

No significant relationships.

